# Unplanned Healthcare Utilization After Emergency Laparotomy: A Systematic Review and Meta‐Analysis

**DOI:** 10.1002/wjs.70355

**Published:** 2026-04-08

**Authors:** Lív í Soylu, Maria Vestergaard, Talha Malik, Jakob Burcharth, Dunja Kokotovic

**Affiliations:** ^1^ Emergency Surgery Research Group Copenhagen (EMERGE) Department of Hepatic and Gastrointestinal Diseases Copenhagen University Hospital—Herlev and Gentofte Herlev Denmark; ^2^ Department of Clinical Medicine University of Copenhagen Copenhagen Denmark; ^3^ Surgical Center of Innovation and Research in Slagelse (SCOLARIS) Region Zealand Slagelse Hospital Slagelse Denmark

**Keywords:** emergency laparotomy, high‐risk surgery, outcomes, readmission

## Abstract

**Background:**

Readmissions following emergency laparotomy are frequent, and mainly a result of late‐onset postoperative complications, worsening of pre‐existing comorbidities, and non‐compliance with care instructions. This review aimed to describe current evidence on readmission after emergency laparotomy.

**Methods:**

A systematic search was performed in MEDLINE, Embase, and the Cochrane Library. Studies were included if they reported readmission incidence following non‐trauma emergency laparotomy for perforated viscera, intestinal obstruction, mesenteric ischemia, or intraabdominal bleeding in adults. Bias was assessed with The JBI Checklist for Prevalence Studies, and certainty of the evidence was assessed using GRADE. The primary outcomes were all‐cause hospital readmission and emergency department utilization within 30‐, 90‐, and 180 days. Secondary outcomes were risk factors and leading causes for readmission.

**Results:**

In total, 78,387 patients (10 studies) were included. Three studies reported on emergency department utilization, and the remaining reported on hospital readmission. The pooled 30‐day hospital readmission rate for 1907 patients was 17% (95% confidence interval (CI) = 16–19%; prediction interval (PI) = 15–20%), and the pooled 30‐day emergency department utilization for 2004 patients was 28% (95% CI = 18–40%; PI = 12–53%). Two studies reported discharge disposition as an independent risk factor of hospital utilization. The leading causes of hospital utilization were wound‐related, abdominal complaints, dehydration and infections.

**Conclusions:**

The cumulative incidence of 30‐day hospital readmission following emergency laparotomy was 16%–19%. These high readmission rates raise global concern, as they may strain hospital resources and compromise quality of care for all patients in need of urgent care.

## Graphical abstract

Readmissions following emergency laparotomy are frequent, and mainly a result of late‐onset postoperative complications, worsening of pre‐existing comorbidities, and non‐compliance with care instructions. This review aimed to describe current evidence on readmission after emergency laparotomy. The cumulative incidence of 30‐day hospital readmission following emergency laparotomy was 16%–19%. These high readmission rates raise global concern, as they may strain hospital resources and compromise quality of care for all patients in need of urgent care.

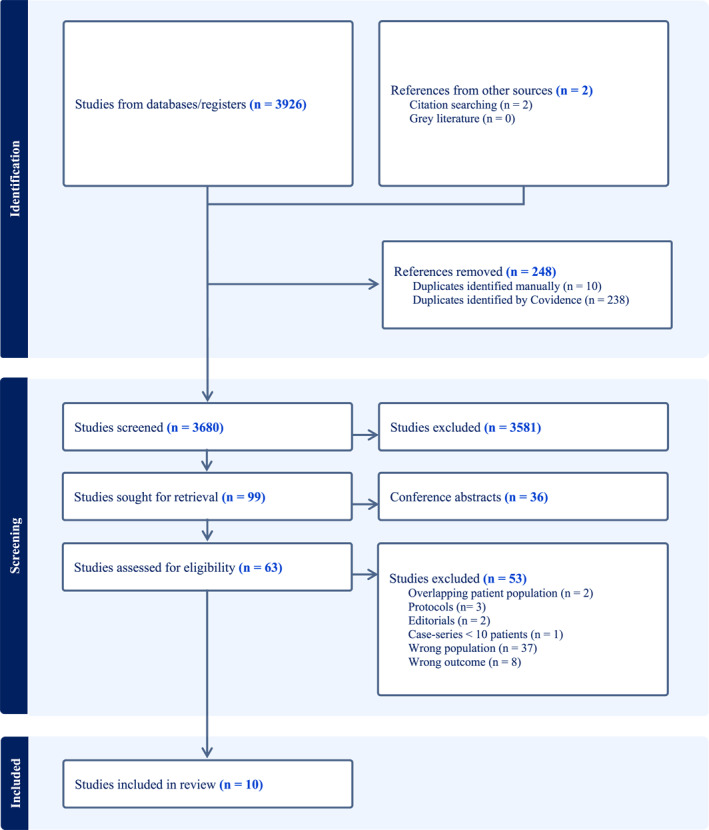

## 1 Introduction

Patients undergoing non‐trauma emergency laparotomy for perforated viscera, bowel obstruction, intestinal ischemia and intraabdominal bleeding are a distinct surgical population with a considerably high risk of mortality and morbidity [1, 2]. The patients are predominantly elderly and suffer from concurrent medical issues, contributing to a reduced physiological reserve [2, 3]. This, in combination with the underlying critical illness and the laparotomy‐inflicted trauma, is prompting an excessive inflammatory response leading to immunosuppression and accelerating breakdown of muscle tissue [4]. Consequently, postoperative patients have a vulnerability for infections and exacerbation of pre‐existing conditions, ultimately leading to an overall health deterioration, resulting in increased healthcare utilization rates [4, 5, 6].

Readmission is a recognized quality of treatment indicator in healthcare, and used as a key metric to monitor hospital performance [7, 8]. In addition, emergency readmissions impose a significant financial burden, and in the US the estimated annual cost of all‐cause 30‐day readmission is exceeding $50 billion [9]. As healthcare costs continue to rise, reducing readmissions presents a strong economic incentive [10]. This has prompted the implementation of readmission reduction programs in several high‐income countries [11].

This study aimed to map the current literature on unplanned hospital contacts following major emergency abdominal surgery, including cumulative incidence, risk factors, and clinical reasons for readmissions until 180 days after surgery.

## 2 Method

This systematic review of incidence was conducted as recommended by the Prevalence Estimates Reviews—Systematic Review Methodology Group (PERSyst) [12, 13]. The Preferred Reporting Items for Systematic Reviews and Meta‐analyses statement was applied [14]. The protocol was registered in the PROSPERO database ID: CRD42024613834. No approvals were required for this study.

### 2.1 Eligibility Criteria

This review was performed according to the following PICO(S): The population (P) comprised adult patients (≥ 18 years of age) undergoing non‐trauma emergency laparotomy for acute intraabdominal pathology (e.g., perforation, obstruction, ischemia, or intraabdominal sepsis), consistent with the definition used by the National Emergency Laparotomy Audit (NELA), which defines emergency laparotomy based on the acute surgical context rather than intraoperative findings or procedural codes [15]. As this review examines the incidence of readmission following emergency laparotomy in patients who receive conventional follow‐up, with no comparative (C) group. The primary outcome (O) was all‐cause “unplanned hospital readmission” and “unplanned emergency department utilization” within 30, 90, and 180 days from discharge or surgery. Secondary outcome measures were risk factors of readmission and leading reasons for readmissions. The study (S) types of interest were randomized clinical trials and observational studies.

Studies were excluded if the cohort comprised patients younger than 18 years of age and laparotomies due to trauma, non‐gastrointestinal etiology (gynecological, urological, and non‐mesenteric vascular etiology), and due to diseases in the appendix or gallbladder regardless of severity, without the possibility of extracting the target population. Furthermore, studies reporting readmission after 180 days from discharge, case series of less than 10 patients, and abstracts with no available full text were excluded. No restrictions were made on language and time of publication.

### 2.2 Search Strategy and Study Selection

On December 10, 2024, a systematic literature search was conducted in MEDLINE, Embase, and the Cochrane Library. A professional medical research librarian from The University of Copenhagen assisted with the search strategy. In our search, we explored the three core components: “Laparotomy”, “emergency”, and “admission”. An exhaustive search was conducted deliberately to avoid excluding relevant studies (Supporting Information S1: Appendix 1). After including relevant studies from the systematic search, a snowball search of reference lists was performed [16].

After the initial searches, records from the three databases were imported to Zotero, (version 6.0.37) and subsequently transferred to a management software (Covidence, 2025), where duplicates were removed [17].

Title and abstract screening were performed independently by LS and MVJ or TM. When deemed eligible, abstracts were passed on for full‐text screening, which LS and MVJ independently screened. Disagreements were settled by discussion.

### 2.3 Data Collection Process

Extracted data items: Author, country, year, study type, examination period, study characteristics, number of patients, surgical indication, baseline characteristics, start of follow‐up, accounted for mortality, readmission definition, hospital utilization incidence, and if readmission to *any* non‐index hospitals were accounted for, as opposed to *just* the index hospital.

Hospital readmission was defined as any unplanned readmission to a hospital department. Emergency department utilization was defined as any emergency department evaluation, regardless of whether it resulted in readmission or a direct discharge.

For studies with unclear readmission assessments, the corresponding author was contacted to obtain further details.

### 2.4 Bias Assessment

The included studies were bias assessed with the JBI checklist for prevalence studies as currently recommended by PERSyst [18, 19]. With the checklist, bias is assessed in nine domains in which the response options are “yes,” “no,” “unclear,” and “not applicable.”

For this review, studies were classified as having “low risk of bias” when none of the nine domains were rated as “no” or “unclear,” and “moderate risk of bias” when one domain was rated as “no” or “unclear,” and “high risk of bias” when more than one domain was rated as “no” or “unclear”.

### 2.5 Outcome Measure

The outcome measure was the cumulative incidence of all‐cause readmission within 30‐ 90‐ and 180 days from discharge or surgery.

### 2.6 Statistical Analysis

The cumulative incidences of readmission, along with its 95% confidence intervals (Cis), were calculated using the Wilson method [20].

Meta‐analyses were conducted, and a logit transformation was applied to stabilize variance in the incidence measure. A random‐effects model was used to account for between‐study heterogeneity, employing the inverse variance method for weighing due to expected variability across studies. The Hartung‐Knapp adjustment was implemented to provide more robust confidence intervals. To assess heterogeneity, the I^2^ and T^2^ were calculated. Prediction intervals were added to the forest plots, as they better account for heterogeneity in incidence measures [19]. Publication bias was assessed qualitatively as the tests used to assess publication bias are developed in the context of comparative data [19]. Three meta‐analyses were performed: (1) 30‐day hospital readmission from discharge, (2) 30‐day emergency department utilization from discharge, and (3) 30‐day hospital readmission from surgery. All data analyses were performed with R (version 4.4.2).

The Grading of Recommendations Assessment, Development, and Evaluation (GRADE) was used to assess the confidence in the estimate derived from the meta‐analyses [21]. The confidence can be graded down according to (1) within‐study bias, (2) result inconsistency, (3) evidence indirectness, (4) estimate imprecision, and (5) reporting bias. The confidence can be graded up according to (1) estimate magnitude, and (2) linearity. Assessment of plausible bias' is not recommended for incidence measures [22]. Based on the overall assessment, the confidence is graded as “high,” “moderate,” “low,” and “very low”. Of note, observational studies are often regarded as superior in measures of proportions because they are specifically designed to assess incidence. In contrast, a randomized controlled trial (RCT) is primarily intended to examine the effect [22].

## 3 Results

**FIGURE 1:**
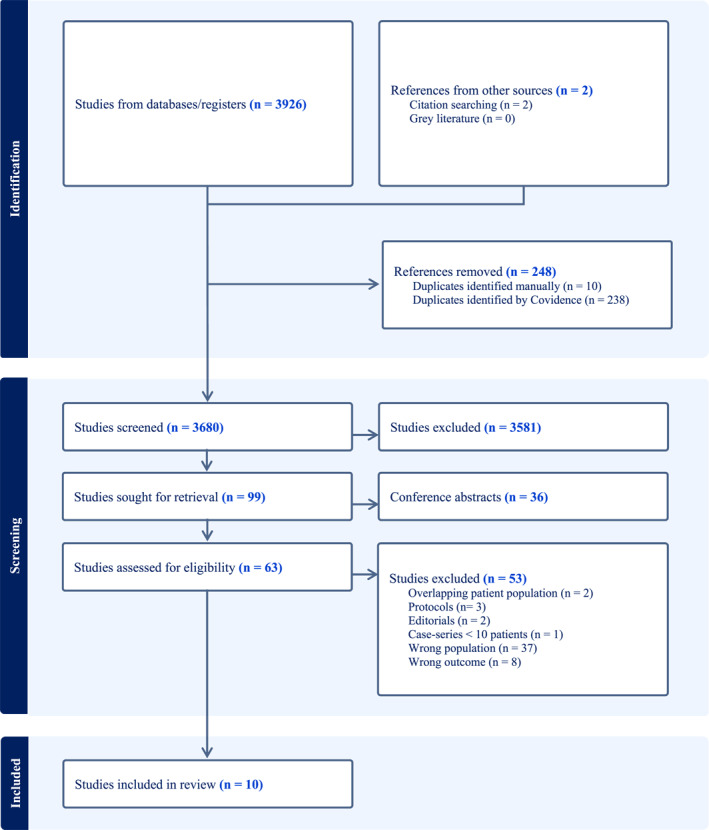
Study selection process.

In total, 3926 studies were identified through the systematic search, and two through other sources. After removing duplicates, 3680 studies were passed on for title and abstract screening, of which 99 were selected for full‐text retrieval. Full‐text was unavailable for 36 abstracts, and they were subsequently identified as conference abstracts without corresponding full‐text publications, leading to their exclusion. Out of the 63 full‐text studies retrieved, three had overlapping populations, of which two were excluded, six were excluded due to the wrong study type, and 37 were excluded due to the wrong study population. Eight were excluded due to the wrong outcome (flowchart in Figure 1). Ultimately, 10 studies were included in the qualitative analysis, of which eight were included in the quantitative analysis.

Three of the selected full‐text studies included patients from the American College of Surgeons National Surgical Quality Improvement Program (ACS NSQIP) within the same inclusion period [23, 24, 25]. All three studies reported on emergency general surgery but included readmission details in subpopulations eligible for this review. However, the subpopulation reported by Nzenwa et al. had the highest compatibility with the aim of this study (Supporting Information S1: Appendix 2) [25].

### 3.1 Study Characteristics

A total of 78,387 patients were included across 10 studies, with the majority being part of a large‐scale study [26]. Among the five studies reporting patient characteristics, 51% were males, 49% were females, and the mean age ranged from 44 to 75 years [27, 28, 29, 30, 31]. The median length of stay of the index admission is reported by seven studies and ranges from 5.5 days to 17 [6, 25, 26, 27, 28, 30, 31]. The operative indications varied: Seven studies examined a mix of high‐risk abdominal conditions [6, 26, 28, 30, 31, 32, 33], two studies examined mechanical obstruction [27, 29], and one study examined outcomes after diverticulitis [25]. Six of the 10 studies reported 30‐day readmission [25, 27, 29, 30, 32, 33], three reported 30‐, 90‐ and 180‐day readmission [6, 26, 31], and one study did not specify the follow‐up time (Table 1) [28]. The studies included patients operated between 2009 and 2022 and were published from 2016 to 2024 (Table 1). One study is set in a lower‐middle income country (India) while the remaining are set in high‐income countries [27].

**TABLE 1:** Baseline characteristics of included studies.

Author Country Year	Study type	Examination period	Original study characteristics	Original cohort (*n*)	Emergency laparotomy indication	Cohort for review (*n*)	Review cohort characteristics	Readmission
Aggarwal India 2023 [27]	Randomized controlled trial	2021–2022	EL^b^ Care pathway 18–85 years	Cases: 30 Controls: 30	Intestinal obstruction	—	Mean age: 46/44 Males: 57/50% ASA^f^ ≥ 3: 30/23% LOS^g^: 5.5 (5–8) /8 (6–11)	30‐day
Barazanchi New Zealand 2022 [28]	Observational retrospective	2012–2017	EL^b^ Sarcopenia ≥ 65 years	167	Mixed high‐risk	—	Mean age: 75 Males: 51% ASA^f^ ≥ 3: 69% LOS^g^: 17 (6–28)	Unclear
Jeppesen Denmark 2016 [29]	Observational retrospective	2009–2013	EL^b^ Morbitity ≥ 18 years	323	Intestinal obstruction	—	Median age: 75 Males: 46%	30‐day
Kongkaewpaisan USA 2021 [30]	Observational prospective	2018–2019	EL^b^ Readmission ≥ 18 years	1347	Mixed high‐risk	—	Median age: 60 Males: 49% LOS^g^: 9 (6–16)	30‐day
Mehta USA 2017 [33]	Observational retrospective	2012–2014	EGS^c^ Hospital volume ≥ 20 years	14,753	Mixed high‐risk	285	—	30‐day
Nzenwa USA 2024 [25]	Observational retrospective^a^	2013–2020	EL^b^ Facial closure ≥ 18 years	Cases: 6217 controls: 30,757	Diverticulitis	Cases: 1688 Matches: 1688	LOS^g^: Cases: 10 (7–16) Matches: 9 (7–14)	30‐day
Í Soylu Denmark 2024 [31]	Observational prospective	2021–2022	EL^b^ Quality of life ≥ 18 years	215	Mixed high‐risk	—	Median age: 66 Males: 52% ASA^f^ ≥ 3: 37% LOS^g^: 8 (5–13)	30‐day 90‐day 180‐day
Í Soylu Denmark 2023 [6]	Observational prospective	2017–2019	MEAS^d^ Readmission ≥ 18 years	504	Mixed high‐risk	442	LOS^g^: 8 (15–4)	30‐day 90‐day 180‐day
Spurling UK 2022 [26]	Observational retrospective	2013–2017	MEAS^d^ DAOH^e^ ≥ 18 years	78,921	Mixed high‐risk	72,084	LOS^g^: 12 (7–21)	30‐day 90‐day 180‐day
Vilches‐Moraga UK 2020 [32]	Observational prospective	2014–2017	EL^b^ Frailty ≥ 75 years	113	Mixed high‐risk	88	—	30‐day

^a^ propensity matched.

^b^ emergency laparotomy.

^c^ emergency general surgery.

^d^ major emergency abdominal surgery.

^e^ validation of Days Alive and Out of Hospital.

^f^ American Society of Anesthesiologists (ASA) physical status classification system.

^g^ Median and interquartile range of index admission length of stay (LOS).

### 3.2 Bias Assessment

After assessment with the JBI checklist, one study was rated with “low risk of bias,” three studies were rated with “moderate risk of bias,” and six studies were rated with “high risk of bias” (Table 2) [25, 27, 28, 29, 32, 33].

**TABLE 2:** Bias assessment with JBI critical appraisal checklist for studies reporting prevalence data.

Author	1. Was the sample frame appropriate to address the target population?	2. Were study participants sampled in an appropriate way?	3. Was the sample size adequate?	4. Were the study subjects and the setting described in detail?	5. Was the data analysis conducted with sufficient coverage of the identified sample?	6. Were valid methods used for the identification of the condition?	7. Was the condition measured in a standard, reliable way for all participants?	8. Was there appropriate statistical analysis?	9. Was the response rate adequate, and if not, was the low response rate managed appropriately?
Aggarwal [27]	No	Yes	Yes	Yes	Yes	Yes	No	Yes	Yes
Barazanchi [28]	No	No	Yes	No	Yes	Unclear	Unclear	Yes	Yes
Jeppesen [29]	No	Yes	Yes	Yes	Yes	No	Unclear	Yes	Yes
Kongkaewpaisan [30]	Yes	Yes	Yes	Yes	Yes	Yes	Yes	Yes	Yes
Mehta [33]	No	Yes	Yes	No	Yes	Yes	Yes	Yes	Yes
Nzenwa [25]	No	Yes	Yes	No	Yes	No	Yes	Yes	Yes
Í Soylu, 2024 [31]	No	No	Yes	Yes	Yes	Yes	Yes	Yes	Yes
Í Soylu, 2023 [6]	Yes	Yes	Yes	No	Yes	Yes	Yes	Yes	Yes
Spurling [26]	Yes	Yes	Yes	Yes	Yes	No	Yes	Yes	Yes
Vilches‐Moraga [32]	No	No	No	Yes	No	Unclear	No	Yes	No

### 3.3 Outcome

The 10 included studies reported a total of 20 outcomes. Nine studies reported 30‐day readmission [6, 25, 26, 27, 29, 30, 31, 32, 33], with two reporting 30‐day hospital readmission and 30‐day emergency department utilization as two separate outcomes [30, 31]. Three studies reported 90‐ and 180‐day readmission [6, 26, 31], with one reporting 90‐ and 180‐day hospital readmission and 90‐ and 180‐day emergency department utilization separately [31]. One study did not specify the follow‐up time for readmission (Table 3) [28].

**TABLE 3:** Readmission assessment and readmission incidence across included studies.

Readmission	Reference	Start of follow‐up	Excluded deceased	Non‐index hospital	Readmission definition	Hospital readmission incidence	Proportion (95% CI)	ED readmission incidence	Proportion (95% CI)
30‐day	Aggarwal [27]	Discharge	Prior to discharge	No	All‐cause	11/60	18.3 (10.6–30.0)	—	—
	Jeppesen [29]	Surgery	Irrelevant	Yes	Related to surgery^b^	56/323	17.3 (13.6–21.8)	—	—
	Kongkaew‐paisan [30]	Discharge	Prior to discharge	Yes	All‐cause	234/1347	17.4 (15.4–19.5)	323/1347	24.0 (21.8–26.3)
	Mehta [33]	Discharge	No	Yes	All‐cause	44/285	15.4 (11.7–20.1)	—	—
	Nzenwa [25]	Surgery	Irrelevant	Yes	All‐cause	402/3376	11.9 (10.9–12.0)	—	—
	Í Soylu, 2024 [31]	Discharge	Prior to discharge	Yes	All‐cause	41/215	19.1 (14.24.4)	61/215	28.3 (22.8–34.7)
	Í Soylu, 2023 [6]	Discharge	Prior to discharge	Yes	All‐cause	—	—	144/442^a^	32.6 (28.4–37.1)
	Spurling [26]	Surgery	Irrelevant	Yes	All‐cause	5574/72,084	7.7 (7.5–7.9)	—	—
	Vilches‐Moraga [32]	Unclear	No	No	Unclear	5/88	5.6 (2.5–12.6)	—	—
90‐day	Í Soylu, 2024 [31]	Discharge	Prior to discharge	Yes	All‐cause	60/215	27.9 (22.3–34.3)	84/215	39.1 (32.8–45.2)
	Í Soylu, 2023 [6]	Discharge	Prior to discharge	Yes	All‐cause	—	—	187/442^a^	42.3 (37.8–47.0)
	Spurling [26]	Surgery	Irrelevant	Yes	All‐cause	13,643/72,084	19.0 (18.6–19.2)	—	—
180‐day	Í Soylu, 2024 [31]	Discharge	Prior to discharge	Yes	All‐cause	73/215	34.0 (28.0–40.5)	97/215	45.1 (38.6–29.3)
	Í Soylu, 2023 [6]	Discharge	Prior to discharge	Yes	All‐cause	—	—	217/442^a^	49.1 (44.5–53.7)
	Spurling [26]	Surgery	Irrelevant	Yes	All‐cause	20,878/72,084	29.0 (28.6–29.3)	—	—
Unclear	Barazanchi [28]	Unclear	No	Unclear	Unclear	29/167	17.3 (13.6–21.8)	—	—

^a^ Information acquired from the author.

^b^ Readmissions deemed “related to the surgery”, without any further clarification.

For five studies, follow‐up for readmission started at discharge [6, 27, 30, 31, 33], for three studies follow‐up started at surgery [25, 26, 29], and in two studies start of follow‐up was unclear [28, 32]. Seven studies assessed all‐cause unplanned readmission [6, 25, 26, 27, 30, 31, 33], one study assessed readmission due to “conditions related to surgery” without further clarification [29], and two studies were unclear about readmission definition (Table 3) [28, 32].

Nine studies reported 30‐day hospital readmission [6, 25, 26, 27, 29, 30, 31, 32, 33], and three reported 30‐day emergency department utilization [6, 30, 31]. Two studies reported 90‐ and 180‐day hospital readmission [26, 31], and two reported 90‐ and 180‐day emergency department utilization [6, 31].

The reported 30‐day hospital readmission rate ranged from 5.6% to 19.1%. When including emergency department utilizations, the rate ranged from 24.0% to 32.6%, with the proportion of treat and release encounters (i.e., emergency department utilization not resulting in hospital readmissions) ranging from 27.6% to 32.8% from the total number of encounters (Table 3 and Supporting Information S1: Appendix 3). The 90‐day hospital readmission rate ranged from 19.0% to 27.9%, and when including emergency department utilization, the rate increased to 39.1%–42.3%. For 180‐day hospital readmission, the rate ranged from 29.0% to 34.0% and from 45.1% to 49.1% when also counting all emergency department utilizations (Table 3 and Supporting Information S1: Appendix 3).

### 3.4 Hospital Readmission Within 30 Days From Discharge

**FIGURE 2:**
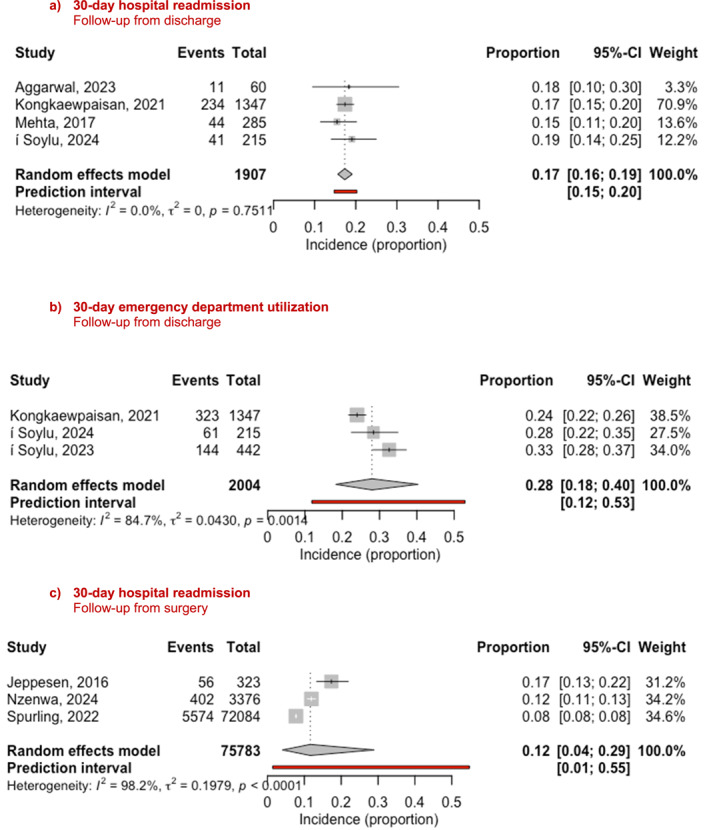
The pooled incidence of 30‐day readmission following emergency laparotomy.

In a meta‐analysis pooling the four outcomes of 30‐day hospital readmission from discharge, the average readmission rate in 1907 patients was 17% (95% confidence interval (CI) = 16–19%; I^2^ = 0.0%; prediction interval (PI) = 15–20%) (Figure 2). The confidence in the estimate was assessed as moderate. According to GRADE, the four studies (one RCT and three observational studies) were initially rated as low quality. However, given the large sample size (*n* = 1907), high consistency (I^2^ = 0.0%), and high precision (PI = 15–20%), the confidence in the evidence was subsequently upgraded to “moderate” (Figure 2 and Supporting Information S1: Appendix 4). A sub analysis including only the three studies from high‐income countries was performed showing similar results (Supporting Information S1: Appendix 5).

### 3.5 Emergency Department Utilization Within 30 Days From Discharge

In a meta‐analysis pooling the three outcomes of 30‐day emergency department utilization from discharge, the average incidence in 2004 patients was 28% (95% CI = 18–40%; I^2^ = 84.7%; PI = 12–53%) (Figure 2). The confidence in the estimate was initially graded as “low” and downgraded to “very low” due to inconsistency (I^2^ = 84.7%) and imprecision (PI = 12–53%) (Figure 2 and Supporting Information S1: Appendix 4).

### 3.6 Hospital Readmission Within 30 Days From Surgery

In a meta‐analysis pooling the three outcomes on 30‐day hospital readmission from surgery, the average readmission rate in 75,783 patients was 12% (95% CI = 4–29%; I^2^ = 98.2%; PI = 1–55%) (Figure 2). The confidence in the estimate was initially graded as “low” and downgraded to “very low” due to immortal time bias (no risk of readmission during index admission), high inconsistency (I^2^ = 98.2%) and low imprecision (PI = 1–55%) (Figure 2 and Supporting Information S1: Appendix 4).

### 3.7 Predictors of Readmission

Two studies reported independent risk factors for readmission in multivariate analyses. One study reported an increased risk of 30‐day hospital readmission in patients with disseminated cancer, 10% weight loss within six months prior to surgery, dyspnea at baseline, wound complications, and discharge to nursing home (Supporting Information S1: Appendix 6 and 7) [30]. The other study reported an increased risk of 30‐ and 180‐day emergency department utilization when discharged with any rehabilitation (in‐home and in‐facility) and in patients with poor quality of life (Supporting Information S1: Appendix 6 and 7) [31].

### 3.8 Readmission Reasons

Two studies reported reasons for readmission: One study reported dehydration (45.5%) and pneumonia (18.2%) as the main reasons for 30‐day hospital readmission in 11 patients [27]. One study reported abdominal complications (37.2%), infections (23.5%), and wounds (11.5%) as the main reasons for 180‐day emergency department utilization in 97 patients (Supporting Information S1: Appendix 6) [31].

## 4 Discussion

This review, examining readmission following emergency laparotomy, included 78,387 patients across 10 studies. The inter‐study readmission assessments were highly heterogeneous, challenging direct comparisons. Nevertheless, in four similar studies (*n* = 1907), the pooled 30‐day hospital readmission rate was 17% (16%–19%), with moderate confidence in the evidence.

Two included studies identified discharge disposition as an independent risk factor for readmission. One study found discharge with rehabilitation to be an independent risk factor for 30‐day emergency department utilization, while another reported discharge to a nursing home as an independent risk factor for 30‐day hospital readmission [30, 31]. These findings are supported by two larger studies in emergency general surgery patients, demonstrating that discharge to any post‐hospitalization care facility independently increases readmission risk [23, 34]. However, these results should be interpreted cautiously, as effect sizes were modest and the outcomes assessed were not identical (emergency department utilization vs. hospital readmission). No additional overlapping risk factors were identified, and the overall evidence remains limited.

Two studies reported the proportion of treat‐and‐release encounters, ranging from 28% to 33% [26, 31]. Such encounters are often considered potentially avoidable. In elective gastrointestinal surgical they are commonly related to wound complications, non‐infectious gastrointestinal complaints, pain complaints, and device issues (e.g., tubes, catheters, ostomies), many of which could potentially be managed in an ambulatory setting [35, 36]. However, the proportion of avoidable *hospital* readmissions following emergency surgery has yet to be reliably determined [37]. In elective general surgery, estimates range from 14.2% (registry data) to 43% (audit of journals) [38, 39]. Given the multifactorial nature of readmission, it has been strongly recommended that “avoidability” should not be determined solely by registry data as this risks underestimation, but rather through a structured peer‐review process [37].

Three studies assessed hospital readmission 30 days from surgery [25, 26, 29]. Spurling et al. reported a 7.7% readmission rate within 30 days of surgery, notably lower than rates measured from discharge [26]. While this approach may be appropriate for low‐risk procedures with same‐day discharge, it may introduce immortal person‐time bias in major surgeries with longer with longer and more variable lengths of stays, potentially explaining the observed differences [40].

Over the past decade, enhanced recovery protocols in emergency laparotomy have reduced mortality and shortened hospital stay [41]. A Danish nationwide study reported increasing readmission rates from 2002–2022 alongside shorter LOS and a decreased mortality suggesting that improved survival and earlier discharge may paradoxically expand the population at risk for readmission [42]. The studies included in this review draw on data from 2009–2022, largely overlapping with the period of widespread enhanced recovery implementation. Moreover, median LOS across the majority of studies ranged between 8 and 10 days, indicating broadly comparable discharge practices and supporting the validity of the pooled estimates.

Beyond temporal changes in surgical care, structural differences in healthcare organization and financial incentives may also influence readmission rates. Some systems impose financial penalties for excess readmissions, others rely on public reporting without direct penalties, and some use bundled payments covering both index admissions and readmissions [10]. These policy differences may affect discharge practices and thresholds for readmission [43]. Broader financing structures further influence access to follow‐up care and thresholds for hospital presentation. Disparities between high‐ and middle‐/low‐income countries in healthcare infrastructure and resources may therefore affect both the occurrence and reporting of readmission and should be considered when interpreting pooled international estimates [43].

There are some limitations to this review. Heterogeneous definitions and measurement approaches complicated inter‐study comparisons; therefore studies were subcategorized according to assessment method for meta‐analysis. Five studies were judged at high risk of bias, primarily due to limited reporting of readmission definitions. Authors were contacted for clarification, although only one responded. Confidence in the evidence ranged from moderate to very low. Although tools such as GRADE were originally developed for comparative data, current recommendations from PERSyst were followed for their adaption to proportion‐based outcomes [12]. Publication bias is also possible, as national or governmental reports may not have been captured in the systematic research.

This review is strengthened by its large sample size and inclusion of 10 studies across diverse countries with varying healthcare systems. The meta‐analysis on hospital readmissions from discharge provides high precision, and the integration of data from India, Denmark, and the US enhances the contextual breadth of the findings. However, structural differences between healthcare systems may contribute to inter‐study heterogeneity and complicate direct comparison. To address this, a supplementary analysis restricted to high‐income countries was performed and demonstrated similar trends, supporting the robustness of the findings.

Readmission is not purely a measure of postoperative quality but a multifactorial outcome shaped by healthcare organization, discharge practices, and patient vulnerability. Patients undergoing emergency laparotomy represent a particularly vulnerable population, often experiencing substantial postoperative morbidity and functional decline, contributing to increased healthcare utilization. Although current knowledge remain limited, available evidence suggests that discharge disposition may serve as an important risk marker for readmission. These findings highlight the need for targeted initiatives for patients suffering from impaired physical functioning, such as optimization of treatment for concurrent medical issues and timely implementation of physiotherapy.

In conclusion, this review offers a comprehensive overview of the current evidence regarding incidence, risk factors and reasons of readmission following non‐trauma emergency laparotomy for high‐risk gastrointestinal emergencies. The estimated 30‐day unplanned hospital readmission rate for the target population is between 16% and 19%, with discharge disposition potentially serving as a key risk factor. To effectively reduce readmission rates, it is crucial to understand not only that readmissions are occurring, but also the underlying reasons behind them.

## Author Contributions

**Lív í Soylu:** data curation, formal analysis, investigation, methodology, writing–original draft. **Maria Vestergaard:** investigation, validation, writing–review and editing. **Talha Malik:** investigation, methodology, validation, writing–review and editing. **Jakob Burcharth:** conceptualization, funding acquisition, methodology, supervision, writing–review and editing. **Dunja Kokotovic:** methodology, supervision, writing–review and editing.

## Funding

The first authors salary was funded by the Novo Nordisk Foundation [NNF22OC0080025]. Otherwise, the project was not funded, and the Novo Nordisk Foundation were not in any way involved in the project.

## Conflicts of Interest

The authors declare no conflicts of interest.

## Supporting information


Supporting Information S1

## Data Availability

All data used in this study is available from the search engines used for the study. The R script for the meta‐analysis can be available upon reasonable request from the first author.
