# Supplementary material for: Unplanned Healthcare Utilization After Emergency Laparotomy: A Systematic Review and Meta‐Analysis
Source: World J Surg. 2026 Apr 8;50(5):1289–99. doi: 10.1002/wjs.70355 (PMC13206593; doi:10.1002/wjs.70355)
Supplement: Supplementary file 1 — Supporting Information S1 [file WJS-50-1289-s001.docx]

**Supplementary material**

Appendix 1 – Search strategy

Appendix 2 – Details on excluded studies

Appendix 3 – Proportion of treat and release encounters from the total number of emergency department (ED) utilizations

Appendix 4 – Study selection for meta-analyses

Appendix 5 – Sub meta-analysis of high-income countries only

Appendix 6 – Risk factors and main reasons for readmission

Appendix 7 – Graphic presentation of risk factor for readmission following emergency laparotomy

**Appendix 1 – Search strategy**

**MEDLINE (pubmed)**

("laparotomy"[MeSH Terms] OR "laparotom*"[Title/Abstract] OR "laparotom*"[All Fields]) AND ("acute care surgery"[MeSH Terms] OR "emergency treatment"[MeSH Terms] OR "emergencies"[MeSH Terms] OR ("emergen*"[Title/Abstract] OR "immediat*"[Title/Abstract] OR "urgen*"[Title/Abstract] OR "acut*"[Title/Abstract])) AND ("patient readmission"[MeSH Terms] OR "hospitalization"[MeSH Terms] OR ("readmiss*"[Title/Abstract] OR "hospitali*"[Title/Abstract]))

Results: 1445 on December 10, 2024

**EMBASE (ovid)**

1. exp laparotomy/
2. laparotom*.mp.
3. exp major surgery/
4. 1 or 2 or 3
5. exp emergency surgery/
6. exp emergency/
7. emergen*.ti,ab.
8. immediat*.ti,ab.
9. urgen*.ti,ab.
10. acut*.ti,ab.
11. 5 or 6 or 7 or 8 or 9 or 10
12. exp hospital admission/
13. readmiss*.ti,ab.
14. rehospitali*.ti,ab.
15. recurring hospitali*.ti,ab.
16. exp hospital readmission/
17. 12 or 13 or 14 or 15 or 16
18. 4 and 11 and 17

Results: 2470 on December 10, 2024

**Cochrane Library**

#1 MeSH descriptor: [Laparotomy] explode all trees

#2 major near/3 surgery

#3 #1 or #2

#4 MeSH descriptor: [Emergencies] explode all trees

#5 MeSH descriptor: [Acute Care Surgery] explode all trees

#6 emergency or urgent or acute or immediate

#7 #4 or #5 or #6

#8 MeSH descriptor: [Patient Readmission] explode all trees

#9 recurring admission

#10 rehospitalization

#11 #8 or #9 or #10

#12 #3 and #7 and #11

Results: 11 on December 10, 2024

**Appendix 2 – Details on excluded studies**

| **Exclusions** | | |
| --- | --- | --- |
| Category | Subcategories | No. |
| **Wrong study type** | | **6** |
|  | Protocols for future study | 3 |
|  | Editorials | 2 |
|  | Sample size < 10 patients | 1 |
|  | | |
| **Wrong patient population** | | **37** |
|  | Patients from 16 years | 2 |
|  | Elective surgery | 6 |
|  | Not possible to isolate target population | 29 |
|  | | |
| **Same patient population in another included study (ACS-NSQIP)** | | 2 |
| Rossi et al, 2020: ACS NSQIP data 2011-2015 (excluded)  Coimbra et al, 2022: ACS NSQIP data 2013-2019 (excluded)  Nzenwa et al, 2024: ACS-NSQIP data 2013-2020 (included) | |  |
|  | | |
| **Wrong Outcome** | | **8** |
|  | No prevalence measure for readmission | 6 |
|  | Readmission after 180 days from surgery and discharge | 2 |

|  | **30-day ED utilization** | | | **90-day ED utilization** | | | **180-day ED utilization** | | |
| --- | --- | --- | --- | --- | --- | --- | --- | --- | --- |
|  | No. of patients with ED utilization | No. patients with treat and release encounters | Treat and release proportion (%) | No. of patients with ED utilization | No. patients with treat and release encounters | Treat and release proportion (%) | No. of patients with ED utilization | No. patients with treat and release encounters | Treat and release proportion (%) |
| Kongkaew-paisan | 323 | 89 | 27.6% | - | - | - | - | - | - |
| Soylu, 2024 | 61 | 20 | 32.8% | 84 | 24 | 28.6% | 97 | 24 | 24.7% |

**Appendix 3 – Proportion of treat and release encounters from the total number of emergency department (ED) utilizations**


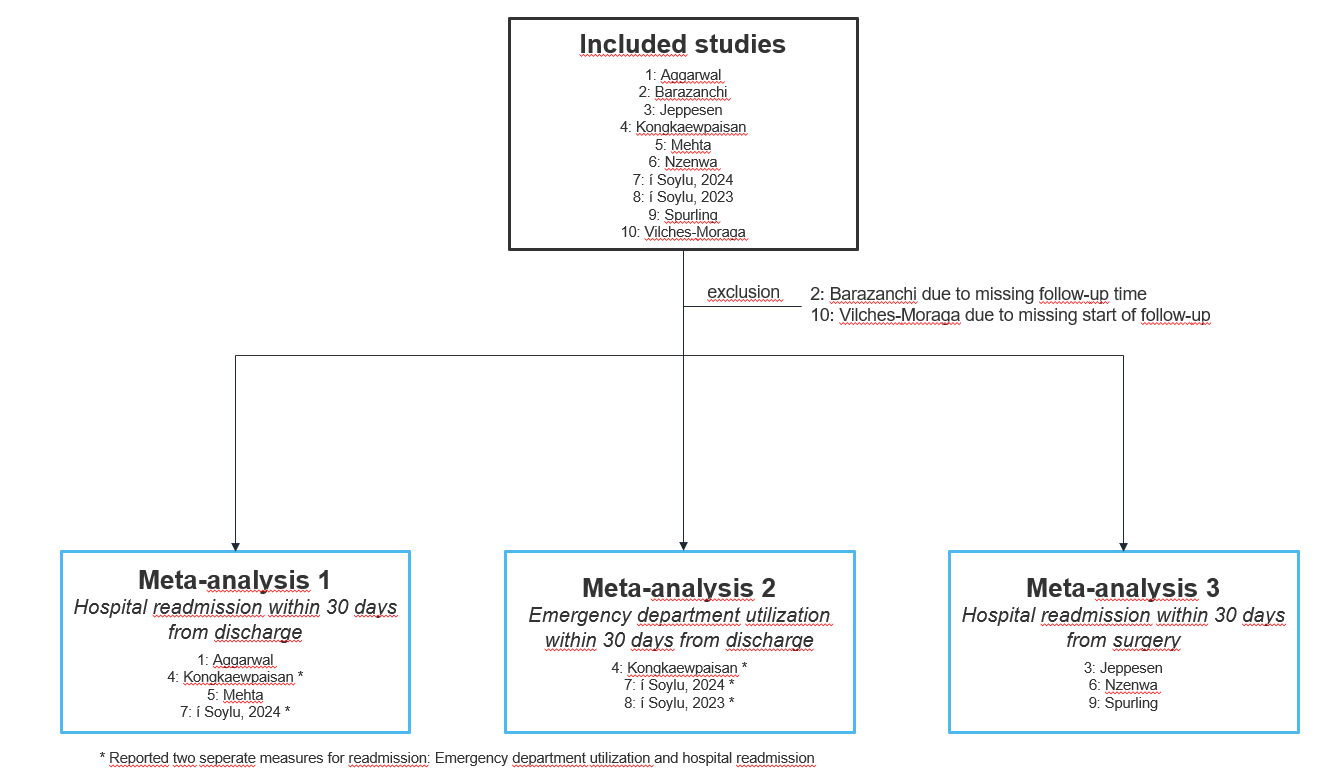
**Appendix 4 – Study selection for meta-analyses**

**Appendix 5 – A subanalysis of meta-analysis 1, including studies from the high income countries only**


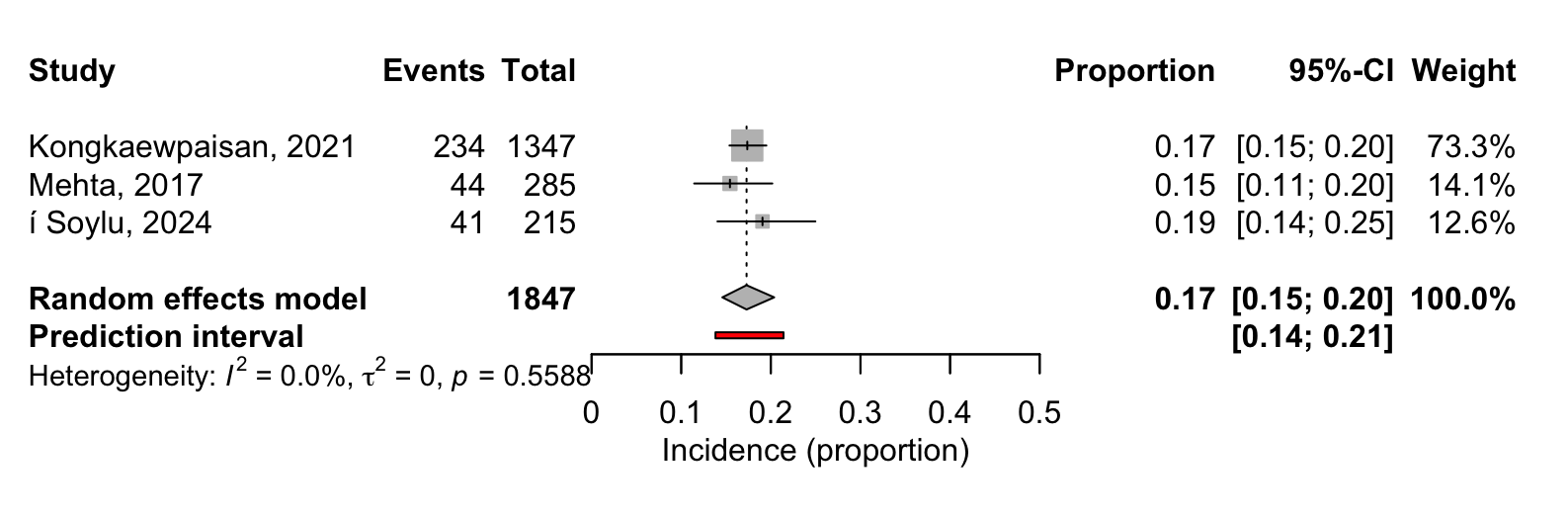


| **Appendix 6 – Risk factors and main reason for readmission** | | | | |  |
| --- | --- | --- | --- | --- | --- |
| **Author** | **Readmission assessment and statistical method** | **Risk factors** | **Results** | **Main causes (%)** | |
| Aggarwal | - | - | - | 30-day hospital readmission  Dehydration (45.5)  Pneumonia (18.2) | |
| Kongkaewpaisan | Observational prospective  Multivariate logistic regression† | Disseminated cancer,  >10% weight loss past 6 months, dyspnea at baseline, discharge to a nursing home and wound complications are independent risk factors for 30-day hospital readmission | Disseminated cancer  2.22 (1.35-3,64), p = 0.002  >10% weight-loss last 6 months  1.65 (1.07-2.54), p = 0.023  Dyspnoe at baseline  1.62 (1.06-2.48), p = 0.026  Wound complications  2.23 (1.55-3.19), p < 0.001  Discharge to nursing home  1.68 (1.02-2.80), p = 0.044 | - | |
| Soylu | Observational prospective  Cox proportional hazard regression* | Low quality of life and discharge to other than own home are independent risk factors for 30 and 180-day emergency department utilization | 30- and 180-day, respectively  Discharge to rehabilitation  2.23 (1.18–4.21), p= 0.014  1.72 (1.04–2.84), p = 0.036  Low quality of life  2.32 (1.06–5.05), p= 0.035  1.99 (1.10–3.63), p= 0.023 | 180-day emergency department utilization  Abdominal complaints (37.2)  Infections (23.5%)  Wounds (11.5) | |

†Adjusted for all demographic, comorbidity, operative, and postoperative variables that have P values <0.02 on univariate analyses

*Adjusted for quality of life (high, intermediate, or low), sex (male/female), age (index procedure (yes/no), any postoperative complication with a Clavien–Dindo classification score ≥2 (yes/ no), and discharge with in‐home assistance or to a rehabilitation facility (yes/no)

**Appendix 7. Graphic presentation of risk factors for readmission following emergency laparotomy**

**
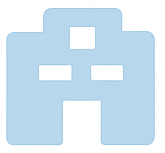

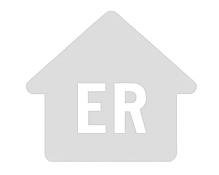
**

**Preoperative**

**Postoperative**

Low health-related quality of life

Difficulty breathing at baseline

Discharge to rehabilitation

(in-home or in

facility)

**Baseline**

**Risk factors**

**30 days & 180 days**

**30 days**

**Emergency department utilization**

**Hospital readmission**

Disseminated cancer

&

10% weight loss < 6 months from surgery

Wound complication

&

Discharge to nursing home
